# CONUT score as a prognostic biomarker in progressive pulmonary fibrosis: a simple tool for clinical risk stratification

**DOI:** 10.1186/s12890-025-04013-0

**Published:** 2025-11-18

**Authors:** Esma Sevil Akkurt, Tugce Sahin Ozdemirel, Berna Akıncı Ozyurek, Kerem Ensarioglu, Ozlem Duvenci Birben, Esma Zenbilli

**Affiliations:** 1 Ankara Oncology Training and Research Hospital, University of Health Sciences, Ankara, Turkey; 2 Ankara Atatürk Sanatorium Training and Research Hospital, University of Health Sciences, Ankara, Turkey

**Keywords:** Progressive pulmonary fibrosis, CONUT score, Prognostic biomarker, Malnutrition, Nutritional risk, Interstitial lung disease, Risk stratification

## Abstract

**Background:**

Progressive pulmonary fibrosis (PPF) is a life-limiting condition characterized by progressive decline in lung function and poor survival. Malnutrition is a frequent but under-recognized factor influencing outcomes in chronic lung diseases. The Controlling Nutritional Status (CONUT) score, derived from serum albumin, cholesterol, and lymphocyte count, may provide a simple prognostic tool in this population.

**Methods:**

In this retrospective cohort study, 58 patients with PPF were evaluated. Patients were categorized into three nutritional risk groups according to their CONUT scores: normal, mild, and moderate malnutrition. Clinical parameters, laboratory findings, pulmonary function decline, and survival outcomes were compared. Kaplan–Meier and Cox regression analyses were performed to assess overall survival and identify independent prognostic factors.

**Results:**

Higher CONUT scores were significantly associated with greater reductions in forced vital capacity (FVC) and increased all-cause mortality. Patients with moderate malnutrition showed significantly worse survival compared to those with normal nutritional status (hazard ratio [HR] = 25.92, 95% CI 1.52–40.65, *p* = 0.024). The CONUT score also correlated with serum cholesterol levels and six-month FVC decline. Steroid use did not significantly affect prognosis.

**Conclusions:**

The CONUT score is a simple, low-cost, and objective biomarker of nutritional risk that may help predict clinical outcomes in patients with PPF. Incorporating nutritional assessment into routine evaluation could improve risk stratification and guide clinical decision-making. Further prospective studies are required to validate its prognostic utility in fibrosing interstitial lung diseases.

## Introduction

Progressive pulmonary fibrosis (PPF) encompasses a spectrum of fibrosing interstitial lung diseases (ILDs) beyond idiopathic pulmonary fibrosis (IPF). According to the 2022 joint guidelines by the American Thoracic Society (ATS), European Respiratory Society (ERS), Japanese Respiratory Society (JRS), and Latin American Thoracic Association (ALAT), approximately one-third of patients with fibrosing ILDs other than IPF may develop a progressive fibrotic phenotype. PPF is characterized by worsening respiratory symptoms, decline in pulmonary function, radiological progression, and premature mortality despite appropriate treatment, often resembling the clinical trajectory of IPF [1, 2].

Irrespective of the underlying etiology, PPF shares common pathogenic mechanisms, including persistent epithelial injury, fibroblast proliferation, and aberrant alveolar remodeling [3, 4]. However, predicting which patients with non-IPF ILDs will evolve into a progressive fibrotic course remains a significant clinical challenge.

Nutritional status has emerged as an important prognostic determinant in chronic respiratory diseases, including IPF. Prior studies have examined indices such as body mass index (BMI), lean body mass, and the Geriatric Nutritional Risk Index. However, indices incorporating BMI may be influenced by racial and body composition differences, limiting their generalizability. Consequently, there is a need for a more practical and universally applicable nutritional assessment tool.

The Controlling Nutritional Status (CONUT) score is a simple and objective screening tool derived from routine biochemical parameters: serum albumin (reflecting protein reserves), total cholesterol (caloric status), and lymphocyte count (immune competence) [5]. CONUT has been validated as a predictor of adverse outcomes in various contexts, including gastrointestinal surgery, cardiovascular disease, end-stage renal disease, and malignancies [6–8].

Although the CONUT score has been widely studied in oncology and internal medicine, its prognostic role in fibrotic lung diseases, particularly PPF, has not been clearly established. This study aimed to evaluate the prognostic utility of the CONUT score in patients with PPF.

## Materials and methods

### Subjects

This retrospective study included 58 patients diagnosed with progressive pulmonary fibrosis who were followed at the Department of Pulmonology, Dr. Abdurrahman Yurtaslan Ankara Oncology Training and Research Hospital, between February 2022 and July 2024. Data were collected from patients within this period, and all patients were followed until July 2024, ensuring a minimum follow-up duration of six months. Patients who refused medical treatment during follow-up or whose radiologic, pulmonary function test (PFT), or survival data were missing from hospital records or patient files were excluded. Clinical data—including comorbidities, laboratory values, PFT parameters at diagnosis, six-minute walk test (6MWT) results, body mass index (BMI), prognosis, treatments, and survival outcomes—were collected retrospectively.

All cases were systematically evaluated through a multidisciplinary discussion (MDD) involving pulmonologists, radiologists, and pathologists, in accordance with international guidelines.

The study was approved by the Ethics Committee of Dr. Abdurrahman Yurtaslan Ankara Oncology Training and Research Hospital (Approval No: 2024-BÇEK/96, dated 15 March 2024), and conducted in accordance with the Declaration of Helsinki (2013 revision). Written informed consent to participate was obtained from all patients at the time of diagnosis and data collection. Clinical trial number: not applicable, as this was a retrospective study.

### Inclusion Criteria

Patients meeting the following criteria were included:

- Age ≥ 18 years,
- Diagnosed with PPF,
- Available demographic, clinical, radiologic, and laboratory data, including exposure history, high-resolution computed tomography (HRCT), pulmonary function and diffusion capacity tests (DLCO), and one-year prognosis data.

### Exclusion Criteria

The following were excluded:

- Incomplete data in hospital records,
- More than one significant comorbidity,
- Presence of active infection at diagnosis.

### Measurements

The CONUT score was calculated using serum albumin, total cholesterol (T-Cho), and total lymphocyte count (T-Lymph), as previously described [7]. Points were assigned based on the degree of reduction: 0–6 for albumin, 0–3 for lymphocyte count, and 0–3 for cholesterol. Scores were classified as: normal (0–1), mild malnutrition (2–4), moderate (5–8), and severe (9–12), with higher scores reflecting poorer nutritional status.

Dyspnea severity was evaluated using the Modified Medical Research Council (mMRC) Dyspnea Scale, ranging from 0 (no dyspnea) to 4 (severe dyspnea limiting daily activity) [9]. Pulmonary function testing included forced vital capacity (FVC), forced expiratory volume in one second (FEV₁), FEV₁/FVC ratio, and diffusion DLCO, in accordance with ATS/ERS standards [10].

According to updated clinical guidelines, PPF is diagnosed in patients with fibrotic ILD (excluding IPF) who demonstrate at least two of the following within the past year without an alternative explanation: (1) worsening respiratory symptoms, (2) physiological evidence of progression, and (3) radiologic progression. All patients with fibrotic nonspecific interstitial pneumonia (F-NSIP) and fibrotic hypersensitivity pneumonitis (F-HP) met the respective ATS and ATS/JRS/ALAT diagnostic criteria.

### Statistical Analysis

Statistical analyses were performed using IBM SPSS version 25.0. Descriptive data were expressed as frequencies (n), percentages (%), means ± standard deviation (SD), and medians (Q1–Q3). Categorical variables were compared using Pearson’s chi-square or Fisher’s exact test. Survival analysis was performed using the Kaplan–Meier method, and Cox regression analysis was applied to identify prognostic factors. A* p-*value < 0.05 was considered statistically significant.

## Results

A total of 58 patients with PPF were included. Of these, 32 (55.2%) were male and 26 (44.8%) were female. Only 6.8% of patients had no comorbidities, with hypertension being the most frequent (48.2%). The most common presenting symptoms were dyspnea (69.0%) and cough (31.0%). Antifibrotic therapy was administered to all patients (nintedanib in 82.8%, pirfenidone in 17.2%), while 44.8% received corticosteroid treatment. The most frequent treatment-related adverse effects were photosensitivity (10.3%), diarrhea (13.7%), elevated liver enzymes (6.8%), weight loss (6.8%), weakness (6.8%), and nausea/vomiting (3.4%).

Among PPF subtypes, F-HP was the most common (48.2%), followed by F-NSIP (34.4%), connective tissue disease-associated ILD (CT-ILD, 10.3%), and sarcoidosis (6.9%). The majority of diagnoses (79.3%) were made based on clinical and radiological findings without histopathological confirmation.

At six-month follow-up, FVC declined in 44.8% of patients, remained stable in 37.9%, and improved in 17.2%. Hospitalization occurred in 69.0% of patients during the follow-up period. Mild desaturation was observed in 48.3% of patients, while 44.8% exhibited severe desaturation. The overall all-cause mortality rate was 17.2%. Baseline clinical characteristics are summarized in Table 1.

**Table 1:** Baseline demographic and clinical characteristics of the study population (*n* = 58)

Variable	*n* (%)/Median (IQR)
Age (years)	70 (64.5–74.5)
Gender – Male	32 (55.2)
Gender – Female	26 (44.8)
Comorbidities	
• Hypertension	28 (48.2)
• Diabetes mellitus	24 (41.3)
Symptoms	
• Dyspnea	40 (69.0)
• Cough	18 (31.0)
PPF Subtypes	
• Fibrotic HP	28 (48.2)
• Fibrotic NSIP	20 (34.4)
• CTD-ILD	6 (10.3)
• Sarcoidosis	4 (6.9)
Diagnosis method	
• Radiology only	46 (79.3)
• Radiology + Pathology	12 (20.7)
Treatment	
• Nintedanib	48 (82.8)
• Pirfenidone	10 (17.2)
• Corticosteroids	26 (44.8)
Hospitalization ≥ 1	40 (69.0)
Mortality	10 (17.2)

The median CONUT score was 1 (range 0–5). Nutritional status was classified as normal in 69.0% of patients, mild malnutrition in 27.6%, and moderate malnutrition in 3.4% (Table 2).

**Table 2:** Distribution of CONUT scores in patients with PPF

Variable	Values
Median CONUT score (min–max)	1 (0–5)
Degree of malnutrition	
*• Normal*	40 (69.0)
*• Mild*	16 (27.6)
*• Moderate*	2 (3.4)

When stratified by CONUT score, patients with mild or moderate malnutrition demonstrated a significantly greater decline in FVC at six months compared with those with normal nutritional status (*p* = 0.017). Median cholesterol levels were also significantly lower in the mild malnutrition group (*p* = 0.015). Mortality was significantly higher among patients with mild malnutrition (37.5%) and moderate malnutrition (100%) compared to those with normal nutritional status (5%) (*p* = 0.010) (Table 3).

**Table 3:** Comparison of disease characteristics of patients according to CONUT score

Variable	Normal (*n* = 40)	Mild (*n* = 16)	Moderate (*n* = 2)	*p*-value
Age (years)	70 (62–78)	67.5 (54–80)	75	0.366
Male sex, n (%)	18 (45.0)	14 (87.5)	0 (0.0)	0.066
6-month FVC decline, n (%)	12 (30.0)	12 (75.0)	2 (100.0)	0.017*
Cholesterol (mg/dL)	191.5 (144–256)	145.5 (109–258)	170	0.015*
Albumin (g/L)	39.7 (36–46)	38.0 (29.1–43.0)	28.7	0.082
Lymphocyte count (×10⁹/L)	2.23 (1.29–5.03)	2.46 (1.34–4.00)	2.37	0.817
Mortality, n (%)	2 (5.0)	6 (37.5)	2 (100.0)	0.010*

*Data are presented as median (range) or n (%). *p* < 0.05 considered statistically significant

Logistic regression analysis did not demonstrate a significant association between CONUT scores and FVC decline (*p* > 0.05). However, Cox regression analysis revealed that a moderate CONUT score was independently associated with increased mortality risk (HR = 25.92, 95% CI 1.52–40.65, *p* = 0.024). In contrast, corticosteroid use was not significantly associated with survival (*p* > 0.05). These findings are summarized in Table 4.

**Table 4:** Effect of CONUT score and steroid use on survival (Cox regression analysis)

Variable	Category	Median survival (days)	HR (95% CI)	*p*-value
CONUT score	Normal*	981	1.00 (reference)	–
	Mild	810.5	8.14 (0.85–78.37)	0.070
	Moderate	583	25.92 (1.52–40.65)	0.024
Steroid use	No*	933.5	1.00 (reference)	–
	Yes	925	5.18 (0.58–46.36)	0.141

*Reference category. Cox regression analysis; *p* < 0.05 considered statistically significant

**Fig. 1:**
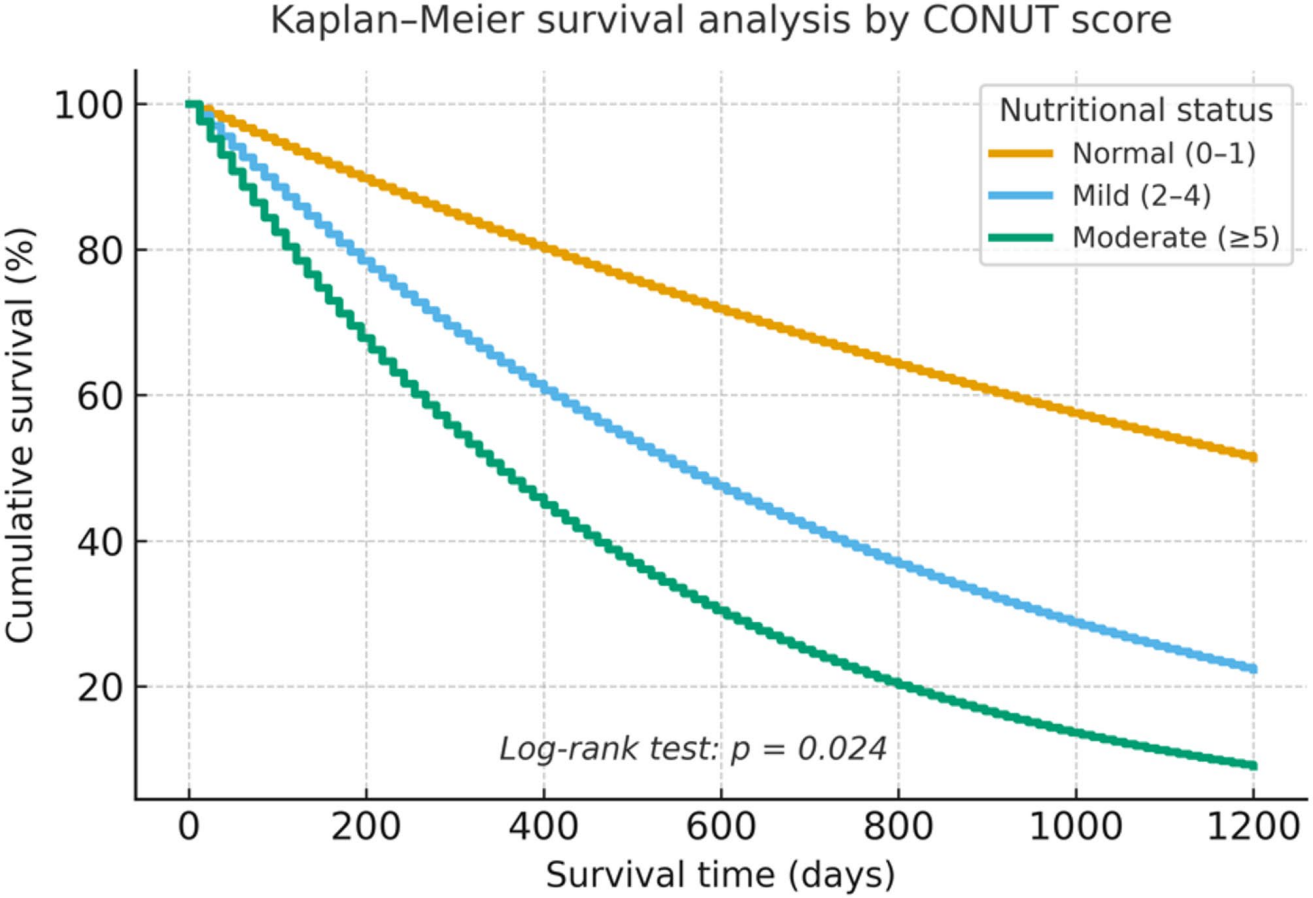
Kaplan–Meier survival analysis according to CONUT score categories. Censored observations are indicated by tick marks. Group separation: Normal (0–1), Mild (2–4), Moderate (≥5). Log-rank test, *p*= 0.024.

Kaplan–Meier survival analysis demonstrated significant survival differences among CONUT categories (log-rank *p* = 0.024). Patients with moderate malnutrition showed the shortest survival, whereas those with normal nutritional status had the most favorable outcomes (Fig. 1). Censored cases were appropriately indicated on the survival curves.

## Discussion

This study aimed to evaluate the prognostic value of the CONUT score in patients with PPF. Our findings demonstrated that a moderate CONUT score was significantly associated with worse survival outcomes. Malnutrition, which can result from increased respiratory muscle workload, systemic inflammation, hypoxemia, and physical inactivity, is a well-recognized factor influencing outcomes in chronic respiratory diseases, including PPF [11].

While BMI has been frequently used to assess nutritional status, its limitations due to ethnic and regional variability reduce its predictive power. For instance, Nakatsuka et al. found an association between annual weight loss and mortality in Japanese patients, but baseline BMI had no significant prognostic value [12]. Similarly, in our study, BMI was not identified as a prognostic indicator.

Previous cohort studies have described the demographic and etiologic characteristics of PPF populations. Compared to those, our study cohort had a higher median age (70 years) and a male predominance (55.2%). F-HP was the most common subtype (48.2%), followed by F-NSIP (34.4%) and CTD-ILD (10.3%). These distributions differ slightly from other published cohorts, which often report autoimmune ILD or CTD-ILD as the predominant etiology [13–16].

The CONUT score, calculated using serum albumin, total cholesterol, and lymphocyte count, offers an objective and accessible method for evaluating nutritional status. Each component has independent prognostic relevance: albumin as a marker of protein reserves, lymphocyte count as an indicator of immune capacity, and cholesterol reflecting caloric status [17]. A study by Iwanami et al. reported that moderate CONUT scores were associated with increased all-cause mortality in patients with IPF [18]. In our PPF cohort, 27.6% of patients were mildly malnourished and 3.4% moderately malnourished, with the latter group demonstrating significantly worse survival. Kaplan–Meier analysis in our study further illustrated the survival gap between nutritional categories.

The interplay between malnutrition, systemic inflammation, and declining pulmonary function plays a critical role in disease progression and mortality in chronic lung diseases [19]. In our study, patients with mild malnutrition exhibited significantly greater reductions in FVC at six months compared with those with normal nutritional status. Although logistic regression analysis did not show a significant association between CONUT score and functional decline, Cox regression analysis revealed that a moderate CONUT score independently predicted mortality. The wide confidence intervals observed, however, highlight potential model instability due to the small sample size, and the results should be interpreted cautiously. These findings are consistent with previous reports linking low albumin levels and nutritional deficits to poor outcomes in PF-ILD, and suggest a potential role for nutritional support in disease management [19, 20].

The prognostic relevance of the CONUT score has also been reported in other chronic respiratory diseases. Lo Buglio et al. demonstrated that higher CONUT scores were associated with more frequent exacerbations in COPD [21], and additional studies have shown its predictive value for cardiovascular disease and mortality in COPD patients [22]. Collectively, these findings support the broader applicability of the CONUT score across chronic lung diseases, including PPF [23].

Several limitations must be acknowledged. First, as a retrospective single-center study, generalizability is limited, and residual confounding cannot be excluded. Second, the relatively small and heterogeneous sample size, with only two patients in the moderate malnutrition group, reduced the statistical power and may have contributed to unstable regression estimates. Third, data regarding statin use, which may influence cholesterol levels and thereby affect the CONUT score, were not available. Finally, although this study adds to the limited literature on nutritional assessment in PPF, similar analyses have been reported in IPF and PF-ILD cohorts; therefore, our results should be interpreted as complementary rather than entirely novel. The relatively small sample size and retrospective, single-center design may limit the generalizability of our findings; therefore, prospective validation in larger cohorts is warranted.

## Conclusion

Malnutrition is a critical yet often overlooked factor influencing the prognosis of patients with PPF. The CONUT score—a simple, objective, and accessible nutritional assessment tool—was associated with survival in this population. Given the potential impact of nutritional status, integrating individualized nutritional interventions alongside antifibrotic therapies may provide a more comprehensive management approach. Future prospective, multicenter studies are warranted to validate these findings and to determine the potential benefits of nutritional support in patients with PPF.

## Data Availability

The datasets generated and/or analyzed during the current study are available from the corresponding author on reasonable request.
